# Social and Psychological Predictors of Body Mass Index among South Africans 15 Years and Older: SANHANES-1

**DOI:** 10.3390/ijerph16203919

**Published:** 2019-10-15

**Authors:** Zandile June-Rose Mchiza, Whadi-Ah Parker, Muhammad Zakir Hossin, Amy Heshmati, Demetre Labadarios, Daniel Falkstedt, Ilona Koupil

**Affiliations:** 1 School of Public Health, University of the Western Cape, Bellville 7535, South Africa; 2 Social Aspects of Public Health (SAPH), Human Sciences Research Council, Cape Town 8000, South Africa; wparker@hsrc.ac.za; 3 Department of Public Health Sciences, Karolinska Institutet, Stockholm SE-171 76, Sweden; zakir.hossin@ki.se (M.Z.H.); daniel.falkstedt@ki.se (D.F.); ilona.koupil@su.se (I.K.); 4 Department of Public Health Sciences, Stockholm University, Stockholm SE-106 91, Sweden; amy.heshmati@su.se; 5 Centre for Health Equity Studies, Stockholm University, Stockholm SE-106 91, Sweden; 6 Faculty of Medicine and Health Sciences, Stellenbosch University, Francie van Zijl Drive, Tygerberg 7505, South Africa; dlabadarios@cybersmart.co.za

**Keywords:** body mass index, underweight, overweight, obesity, social determinants of health, inequalities

## Abstract

This study investigated how psychological distress and the proxies for social position combine to influence the risk of both underweight and overweight in South Africans aged 15 years and older. This was a cross-sectional study that included 2254 men and 4170 women participating in the first South African National Health and Nutrition Examination Survey (SANHANES-1). An analysis exploring the associations of social and mental health characteristics with body mass index (BMI) was conducted using binary and multinomial logistic regressions. Results suggested that, overall, women had a higher risk of overweight/obesity compared to men (age-adjusted odds ratio [AOR] 4.65; 95% confidence intervals [CI] 3.94–5.50). The gender effect on BMI was smaller in non-African participants (AOR 3.02; 95% CI 2.41–3.79; *p*-value for interaction = 0.004). Being employed and having a higher level of education were associated with higher risks of overweight and obesity and a lower risk of underweight. Being single or without a spouse and poor mental health were found to increase the odds of being underweight, especially in men. To conclude, there are strong social gradients and important gender and ethnic differences in how BMI is distributed in the South African population.

## 1. Introduction

Unhealthy weight (underweight and obesity) is rife [1,2,3,4], heterogeneous, and uneven across population groups in South Africa [5,6,7]. This is thought to be influenced by a myriad of factors that include socio-demography, psychology, environment, behaviors and genetics [8,9,10,11,12,13,14,15,16]. For the last two decades, as a result of urbanization and globalization, South Africa has been contending with a nutrition transition which has in turn resulted in an epidemiological transition [1,2,3,4].

With the advent of democracy in South Africa, there has been a spike in the migration of people from rural to urban areas in search of opportunities to secure better incomes [17]. These people have undergone changes to their lifestyle that have been characterized by shifts in their dietary patterns from traditional diets to obesogenic diets, as well as a reduction in energy expenditure [18]. These lifestyle changes have impacted their nutritional status, as evidenced in the anthropometric transition in South Africa [3,4]. For instance, while underweight has decreased in all population groups in South Africa in recent years, the overall prevalence of overweight and obesity has increased by 2% in men and 12% in women between 1998 and 2016 (from 29% to 31% in men and from 56% to 68% in women) [1,3]. Moreover, while obesity has increased in all geographic locations, it has increased at a faster rate in urban compared to rural areas. In rural areas, the obesity prevalence increased from 6.0% to 6.8% in men and from 26.9% to 39.2% in women between 1998 and 2016 [1,3]. In urban areas, a larger increase has been shown for the same time period, where the prevalence of obesity increased from 11.0% to 13.3% in men and from 26.4% to 42.2% in women.

The increase in obesity in South Africa is a cause for concern given that it is an important risk factor for a number of non-communicable diseases (NCDs) including cardiovascular diseases, type 2 diabetes, musculoskeletal conditions and certain cancers [19,20,21]. According to Patel et al. [22], the higher the increase in body mass index (BMI) prevalence, the higher the probability of developing NCDs such as diabetes and hypertension. In 2000, South Africans who were 30 years and older and who presented with a BMI ≥ 21 kg/m^2^ displayed a fairly high prevalence of the following NCDs: Type 2 diabetes (87%), hypertensive disease (68%), endometrial cancer (61%), ischemic stroke (45%), ischemic heart disease (38%), kidney cancer (31%), osteoarthritis (24%), colon cancer (17%), and postmenopausal breast cancer (13%) [21].

It is important to remember that while genetics are a strong risk factor for obesity in South Africa [13,14,15], evidence has also highlighted an additional interplay between genetics and other determinants such as sociodemography, especially the social position (also known as social standing) [23], environment, and behaviors [16,17,24,25]. In fact, international evidence has suggested contradictory associations (i.e., both positive and negative) between social position and BMI [7,26,27,28]. It is therefore important that this association is explored in the national South African context.

International evidence has also shown that after age, marital status seems to be the strongest predictor for overweight, obesity, and abdominal obesity in both men and women [29,30,31,32,33]. In fact, Lipowicz et al. [29] showed that married men and women are more likely to be overweight and obese than those who have never been married. Janghorbani et al. [32] showed that the prevalence of overweight was two-fold higher in married men (odds ratio [OR]: 2.24; 95% confidence interval [CI]: 2.08–2.41) and women (OR: 2.36; 95% CI: 2.20–2.53) than never-married men and women, even when age, educational level, leisure time physical activity, smoking habits, and place of residence were controlled for. 

While researchers like Tzotzas et al. [30] confirmed some of the aforementioned hypotheses on BMI and marital status, they also showed that the BMI is inversely associated with education level, especially in women. There is also evidence showing that South Africans who fall below the poverty index line (PIL) (i.e., those who cannot afford to buy a healthy basket of food that offers enough energy and nutrients from food) present with health challenges, among them being overweight and obesity [34,35,36,37,38]. It is therefore important to always consider where South Africans fall in the PIL when explaining their BMI status. 

There is also growing international literature that suggests that BMI is influenced by mental health, body image, and, in particular, psychological distress [39,40,41,42,43]. In fact, Mchiza et al. [39] showed that the majority of South Africans who present with negative body image also tend to present with unhealthy body size (underweight and obesity). Kelly et al. [41], Martinez et al. [42] and Morris [43], on the other hand, showed non-linear relationships between BMI, PIL and psychological distress. Martinez et al. [42], Kim et al. [44] and Kelly et al. [41] showed a U-shaped relationship, while Morris [43] showed a J-shaped relationship between psychological distress and BMI. These studies suggested that when adjusted for age, underweight and overweight individuals presented with elevated odds of being distressed compared to their healthy weight counterparts. In these studies, the middle-aged (40–44 year old) overweight and obese individuals tended to have elevated odds of psychological distress when compared to the younger and older individuals. However, it is important to note that while the relationship between BMI and psychological distress in these studies was modified by age, this interplay was different among women and men. For instance, Brandheim [40] and Morris [43] showed that women tended to have higher psychological distress than men. Brandheim et al. [40] further showed that psychological distress increases with age among women in all BMI categories. However, these authors could not find significant age differences in psychological distress among the different BMI categories of men. It is also important to note that for both genders, any notable increase in psychological distress in the Kelly et al. [41] study was only clearly visible when individuals fell in the class II obesity (i.e., BMI = 35–39.9 kg/m^2^ [45]) category. In South Africa, this kind of literature is scarce. Therefore, it becomes important to explore the relationships between BMI and psychological distress in a representative sample of the South African population with diverse socio-demographic characteristics. 

The current study therefore sought to investigate how psychological distress and the proxies for social position (marital status, education, employment and income statuses) [34,35] combine to influence the risk of both underweight and overweight in South Africans aged 15 years and older. 

## 2. Materials and Methods 

### 2.1. Study Design and Study Population 

This cross sectional study employed secondary analyses of data for 6424 (2254 men and 4170 women) South Africans aged 15 years and older who participated in the first South African National Health and Nutrition Examination Survey (SANHANES-1) [4]. The details of this population selection and recruitment are described elsewhere [39] and are based on the South African population outlined in the 2011 Census report [46]. In summary, of the 8166 households sampled from the detailed procedure presented in Mchiza et al. [39], 8776 eligible individuals who were 15 years or older gave written informed consent to participate in the SANHANES-1. Of these, 6424 were suitable for the analyses, since they had valid and complete measurements for weight and height in order to calculate BMI. These individuals included a larger proportion of female (64.9% females vs. 35.1% males) and African (79.2% Africans vs. 20.1% non-Africans) participants. 

### 2.2. Measures

The interviews were conducted by trained fieldworkers using a previously validated SANHANES-1 questionnaire. The questionnaire contained constructs on participants’ sociodemographic factors (age, gender, ethnicity, and education level, as well as marital, employment and household income statuses) and psychological distress. To assess the socio-demographic factors, participants were given options to choose from 2 categories ([African or non-African] for ethnicity and [employed or not employed] for employment status), 3 categories ([married/cohabiting or never married or divorced/separated/widowed] for marital status and [no household income or low household income or medium to high household income] for income), 4 categories (15–24, 25–44, 45–64, or 65 and above years) for age, and 5 categories (no education or achieved grades 1–7, 8–11, 12, or tertiary education) for education level.

In this analysis, we used the education level, as well as employment, household income and marital statuses as proxies to determine social position. This was done because in South Africa it is difficult to understand the burden of diseases without considering the individuals’ social position, which is determined by the PIL. 

Psychological distress was measured using the Kessler Psychological Distress Scale [47]. This is a 10-item questionnaire intended to yield a global measure of distress based on questions about anxiety and depressive symptoms that a person has experienced in the most recent 4-week period. Scores less than 20, 20–24, 25–29 and greater or equal to 30 indicate mental health wellness, mild mental disorder, moderate mental disorder and severe mental disorder, respectively. For the current analyses, the responses given by the participants were grouped into 2 categories ([No signs of psychological distress or psychological wellness] for scores less than 20 or [Yes, presence of psychological distress] for scores greater or equal to 20).

The participants’ weights and heights were measured based on the Lee and Nieman [48] techniques, and the BMI levels (percentiles for children and kilograms per meter squared [kg/m^2^] for adults) were calculated. Underweight, normal weight (or healthy BMI), overweight, and obesity were defined as BMI levels of <5th percentile and <18.5 kg/m^2^, 5–84.9th percentile and 18.5–24.9 kg/m^2^, 85–94.9th percentile and 25–29.9 kg/m^2^, and ≥95th percentile and ≥30 kg/m^2^ for children and adults, respectively (Centre for Disease Control [CDC], 24/7) [45].

### 2.3. Statistical Analyses

Stata version 15.0 was used for all analyses. We started the analysis by separately investigating the shape and patterns of BMI distribution in men and women and in different age groups. Since the distribution of the BMI was found to be asymmetric, we used it as a categorical variable in the statistical analysis. The age-standardized proportions of BMI categories, together with their 95% confidence intervals (CI), were calculated for all exposure variables. The statistical associations between the exposures and BMI categories were examined in multinomial logistic regression models, with healthy weight used as the reference category. The associations were first assessed in minimally adjusted models, followed by a fully adjusted model for which all exposures of interest were mutually controlled. The estimates were reported as OR with 95% CIs. Since we found some evidence of effect modification by gender, all analyses were separately carried out for women and men. We also explored the possible interactions between each exposure and ethnicity with regard to their effect on BMI categories. As no evidence of effect on heterogeneity was detected, we refrained from stratifying the main analyses on ethnicity. 

Further, we repeated the statistical analysis using binary logistic regression models where BMI was entered as a dichotomized variable (overweight/obesity versus normal weight/underweight). The results from the binary logistic regression were also reported. 

The analyses of social patterning of underweight, overweight and obesity were restricted to 1655 (73%) men and 3104 (74%) women who also had data available on ethnicity, education, marital status, employment and psychological distress. In the analytical subsample of subjects with no missing data, there was an overrepresentation of older, non-African, never married, employed, and participants with a medium level of education. The mean BMI in the analytical subsample was 1.0 (95% CI 0.6, 1.4) kg/m^2^ higher than in the excluded group, and the analytical sample included a higher proportion of overweight and obese subjects. Household income was the variable with highest proportion of missing data, and analyses including household income had to be further restricted to 1140 (50%) men and 2218 (53%) women. We repeated the main analyses of social patterning of BMI in this group to investigate the consistency of findings across the groups with and without data on household income. 

Confidence intervals that did not overlap were used to detect the statistical significance of the associations of interest. *p*-values less than 0.05 were mainly used for the purpose of hypothesis testing. 

Ethics approval to conduct the research was obtained from the Human Sciences Research Council (Reference Number: REC6/16/11/11). All participants 15–18 years had their parents sign informed consent, and the participants gave assent. All adult participants gave written informed consent. 

## 3. Results

### 3.1. Body Mass Index of Participants by Age and Gender 

**Figure 1:**
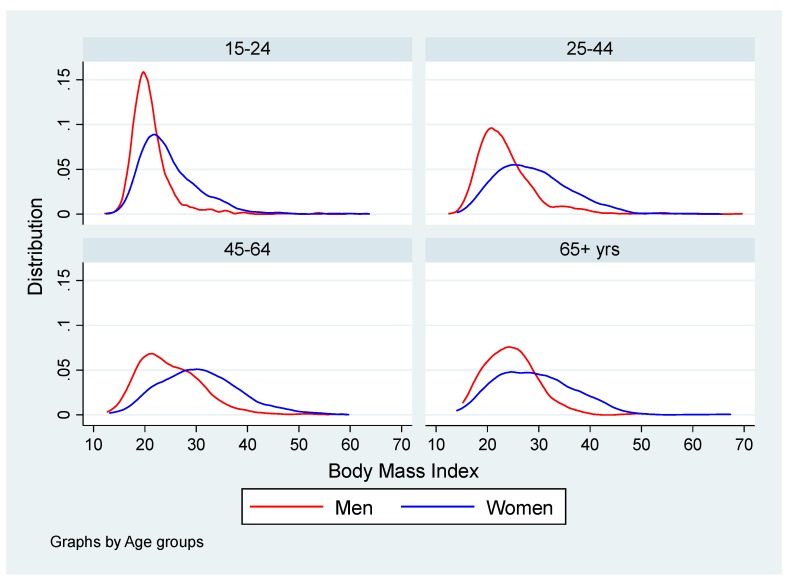
Distribution (kernel density) of body mass index (in percentiles and kilograms per square meters) among 2254 men and 4170 women from the first South African National Health and Nutrition Examination Survey (SANHANES-1) study in South Africa in 2012; by age group.

The participants were 15–98 years old with a mean age of 40 years in men and 41 years in women. Compared to men, the distribution of BMI in women showed greater variation and a higher median value (a median of 27.4 kg/m^2^ in women vs. 22.0 kg/m^2^ in men). There were positively skewed distributions of BMI and strong increases in mean and median BMI with age up to age 65 years in both men and women (Figure 1). 

**Figure 2:**
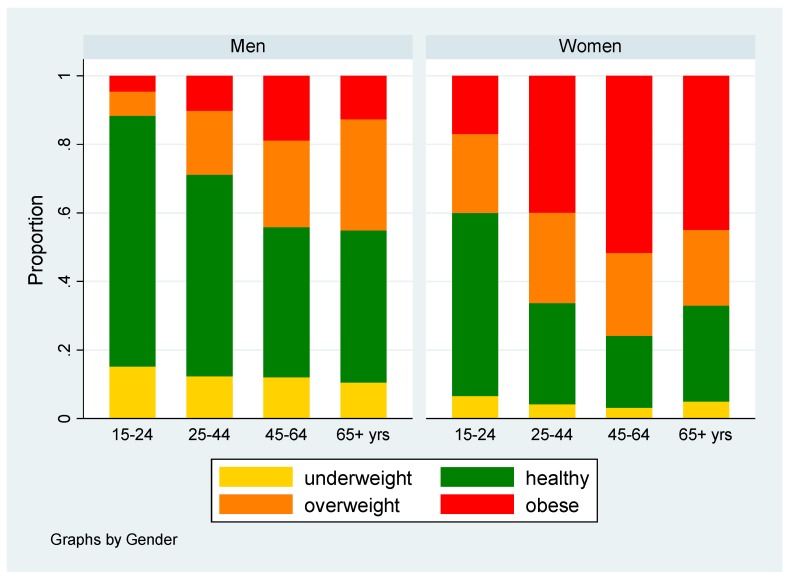
Distribution of body mass index (in percentiles and kilograms per square meters) categories among 2254 men and 4170 women from the SANHANES study in South Africa in 2012; by gender and age.

Less than half of the study population had a healthy BMI (41.5%), and 7.5%, 22.3%, and 28.7% were underweight, overweight and obese, respectively (Table 1). The proportion of subjects with healthy weight decreased and the risk of obesity increased with age up to 65 years in both genders (Figure 2). African women had a particularly high risk of being overweight or obese compared to men (age adjusted OR 4.65; 95% CI 3.94–5.50). The effect of gender was smaller in non-African versus African participants (age adjusted OR 3.02; 95% CI 2.41–3.79; test for statistical interaction *p* = 0.004).

Underweight, healthy BMI, overweight and obesity were defined as BMI levels of <5th percentile and <18.5 kg/m^2^, 5–84.9th percentile and 18.5–24.9 kg/m^2^, 85–94.9th percentile and 25–29.9 kg/m^2^, and ≥95th percentile and ≥30 kg/m^2^ for children and adults, respectively (CDC, 24/7) [45].

In addition to the generally lower proportion of underweight and healthy weight among women compared to men, there were also systematic gender differences in how different aspects of social position were associated with weight categories. Age-adjusted proportions of underweight, healthy weight, overweight and obesity for men and women are shown separately in Table 2 and Table 3.

### 3.2. Proportion of Underweight, Healthy Weight, Overweight and Obesity Participants by Socio-demography 

Table 2 shows that there was a high proportion of underweight among men with no education (26.9%) compared to those who were educated. In this case, the significant difference was visible between this group and those men with education level from grade 8 and above. There was also a significantly high proportion of underweight men with symptoms of poor mental health (18.3%) versus those who were psychologically healthy (11.1%). The proportion of underweight was also significantly higher in men who were not employed (15.8%) compared to those who were employed (4.6%). The proportion of underweight was significantly lower (2.7%) in men who secured a medium-to-high income when compared to those who had a low or no income. The proportion of obesity was significantly higher in men who were employed (17.3%) compared to those who were not (9.3%). The proportions of overweight (24.5%) and obesity (15.6%) were significantly higher in men who were married or cohabiting compared to those who were never married. Finally, the proportions of overweight (27.6%) and obesity (23.6%) were significantly higher in men who secured a medium-to-high income when compared to those who had no income or a low income, respectively.

On the other hand, Table 3 shows that the proportions of obesity were significantly higher among African women (41.1%) and those women with tertiary education (50.9%) compared to those who were non-African, not educated, or achieved grades 1–7 and grade 12. Those women who were not employed also had a significantly higher proportion of healthy BMI (33.6%) when compared to those who were employed (22.1%). The proportion of healthy BMI was significantly higher among women who had achieved grade 12 (36.1%) compared to those who achieved tertiary education and those who achieved grades 8–11. Finally, the proportion of underweight was significantly lower among women who had achieved grades 8–11 (2.8%) compared to those who had no education or achieved grades 1–7.

### 3.3. Multinomial Logistic Regression Analysis of BMI Categories Versus Exposures Using Healthy Weight as the Baseline

Multinomial logistic regressions with age adjustment provided further evidence that employment and higher education were associated with a higher risk of overweight and obesity and a lower risk of underweight in both men and women (Table 4 and Table 5). A lower risk of obesity was seen among non-African compared to African women, and a lower risk of underweight was observed for non-African compared to African men. Poor mental health was also associated with a higher risk of underweight among men (Table 4). In women (Table 5, Model 2), the results were similar when we adjusted the analysis by age only, and when we adjusted the analyses by socioeconomic and socio-demographic factors as well as mental health. In men (Table 4, Model 2), a further adjustment for socioeconomic and health characteristics led to a slight attenuation of the effect sizes, but all the reported associations of ethnicity, education and employment with weight status remained statistically significant. For both genders (Table 4 and Table 5), we found weak evidence of poor mental health being associated with an unhealthy BMI and a significantly higher risk of underweight, specifically in men. There was a statistically significant interaction of gender with ethnicity (*p* = 0.039), education (*p* < 0.001), employment (*p* = 0.012) and marital status (*p* = 0.001) in their effect on weight categories (when Table 4 and Table 5 were combined, shown in the footnote of both tables). 

### 3.4. Restricted Analyses of Social Patterning of Underweight, Overweight and Obesity Versus Household Income 

The associations of household income with weight status were studied in a restricted sample of 1140 men and 2218 women with data available. In men, a higher income was associated with a higher prevalence of overweight and obesity in the age-adjusted as well as fully adjusted model (Table 6). However, there was no significant association between BMI and income observed in women. A test for statistical interaction between income and gender in their effect on weight indicated an effect modification with weaker or non-existent associations between income and weight categories in women (*p*-value interaction 0.042, as shown in the footnote of Table 6). 

### 3.5. The Statistical Analyses Using Binary Logistic Regression Models Where BMI Was Entered as A Dichotomized Variable 

Analyses of BMI categorized as a binary outcome (overweight/obesity vs. normal/underweight) provided results consistent with the associations found in multinomial logistic regressions. We confirmed a statistical interaction of gender with ethnicity, education and marital status in their effect on weight (*p*-value for interaction gender and ethnicity *p* = 0.004, gender and education *p* < 0.001, and gender and marital status *p* = 0.003) (as shown in the footnote for Table 7). Education was more strongly associated with the risk of overweight/obesity in men compared to women. Men with highest education level had a more than four times higher probability of being overweight or obese compared to men with no education (Table 7). 

## 4. Discussion

In this secondary data analysis, we sought to investigate, for the first time, how psychological distress and social position were associated with the development of unhealthy weight in a representative sample of South Africans aged 15–98 years. 

The findings of the current research corroborate previous literature in South Africa [1,2,3] that suggests that underweight is common in men and obesity is high in women. In the current study, while the prevalence of underweight in men was almost four times that of women (4.2% women vs. 11.9% men); the prevalence of obesity in women was almost four times that of men (11.6% men vs. 40.1% women). The findings of the current study also showed that despite the greater variation in BMI levels between men and women, the BMI mean values were higher than the median values in both genders, thereby showing BMI distributions that were skewed to the right. This suggested that the majority (51%) of the South Africans tended to fall within the overweight side of the BMI spectrum. The distribution of South Africans with healthy BMI (20–24.9 kg/m^2^) in men and women also showed that fewer middle-age and older-aged South Africans (25+ and 45+ years, respectively) have a healthy weight when compared to their younger counterparts (<25 years and <45 years, respectively). 

The findings of the current study also showed that poor mental health increases the odds of underweight in both men and women. On the other hand, the association between never being married and underweight did not reach statistical significance in either gender. The association of marital status with overweight and obesity remained significant when other socio-demographic variables were held constant. Moreover, on investigating the other proxies used for social position, it was observed that being educated, employed and having high income were associated with a decreased likelihood of being underweight and an increased likelihood of being overweight and obesity in both genders. These associations remained visible even after removing the confounding effects of the other socio-demographic variables investigated in the current paper. 

On trying to explain the outcomes relating to the marital status in South Africa, we could relate these findings to those of other studies conducted in smaller samples of South Africans. Nienaber-Rousseau et al. [9] for instance, recently suggested that in the African adult Tswana-speaking South African population, being married increased the likelihood of weight gain and adiposity, especially around the waist by 27% in men. Other international studies undertaken in Iran, Greece and Poland undertaken by Janghorbani et al. [32], Tzotzas et al. [30], Lipowicz et al. [29], respectively, also corroborated the relationship between weight gain and marital status. Similar to these international studies, our study also detected a strong association between marital status and overweight/obesity in both genders. In addition, we found that this association was more strongly pronounced in men than in women. The association of marital status with overweight and obesity in both men and women, and a particularly stronger association in men in South Africa as observed in the current study, may be explained by the hypothesis presented in a review of international studies by Dinour et al. [49]. This review suggested that weight gain after marriage or cohabitation tends to occur because of increased opportunities for eating due to couples sharing regular meals characterized by larger portion sizes. 

Indeed, in South Africa, married women are expected to cook and dish up large food portion sizes with an abundance of red meat for their spouses [50]. In fact, in South Africa, there is additional evidence to suggest that having a bigger body size is an indication of beauty and fertility in women and prestige and happiness in both genders [50,51]. Moreover, this evidence further suggests that an overweight married individual is well cared for by their spouse [50,51]. In their review, Dinour et al. [49] further showed that married individuals reduce their participation in physical activity because their focus is taken away from body size maintenance. This is due to the fact that married individuals are no longer looking to attract intimate partners. Married individuals are also less likely to smoke and are more likely to quit smoking [49]. In this review, smoking cessation was associated with weight gain [49]. According to Averett et al. [33] there are four hypotheses that might explain the relationship between marital status transitions and changes in BMI. These hypotheses suggest that (i) leaner individuals are more likely to be selected into marriage, (ii) married individuals present with good health since they get social support from spouses and rarely engage in risky behaviors, (iii) individuals in relationships tend to overconsume energy dense foods due to marriage’s social obligations, and (iv) older adults who are no longer in the marriage market tend not to maintain a healthy BMI because doing so is costly. All these hypothesis may also apply in South Africa; however, testing them is beyond the scope of the current study. 

Other earlier studies conducted by Sobal et al. [52], Eng et al. [53] and Lee et al. [54] on older adults have shown that being unmarried or transitioning out of marriage (especially when individuals get divorced or lose their spouses) is associated with food consumption reduction as a result of changes in social support, social control, stress, and depression. Hence, these individuals tend to present with a reduced body size. Other studies have also associate stress (especially, psychological distress) with age and income [55,56]. For instance, widowers in a study by Wilcox et al. [55] reported substantially higher rates of depression and poorer social functioning. Shahar et al. [56] also found that widowed individuals had less food enjoyment. Despite these topics not being investigated in our research, we found that psychological distress increased the likelihood of underweight in our male participants. However, the origins of mental health deserve closer examination in future studies in South Africa.

Another interesting finding from the current analysis was the interaction between ethnicity and gender, suggesting that being non-African in South Africa increased the likelihood of being overweight and obese in men and reduced this likelihood in women. In fact, in this analysis, more non-African men were overweight and obese than African men, whereas less non-African than African women were overweight and obese. These results are in line with results observed in other national surveys [1,2,3], where more non-African men and more African women have been shown to be overweight and obese. These findings may be explained by the social position proxies in South Africa, where there are distinct education, employment and income statuses between men and women and between African and non-African individuals [57]. In the Statistics South Africa surveys [57], it has been highlighted that while South African women (especially African women [30%]) continue to be the largest group affected by unemployment, 28% of African men are unemployed when compared to 21%, 12% and 8% Colored, Indian/Asian and White men. Of those African men who are employed, the majority are employed in construction industries that require labor-intensive work activities. This therefore increases the likelihood of African men having to expend more energy, a factor that may protect individuals from body fat accumulation [11]. This is a different case with non-African men, the majority of whom are highly educated and have higher incomes since they are employed in high paying jobs. High paying jobs often require individuals to spend most of their time sitting down in meetings or in front of a computer screen. This therefore reduces these men’s chance to expend energy, a factor that fuels body fat accumulation [11]. On the other hand, while the majority of African women are unemployed, the majority of those who are employed spend most of their time around food as domestic workers and workers in food industry, where making and tasting food is the basis of their employment. This therefore increases their chance to overeat; hence, the majority are overweight and obese. 

Finally, when it comes to the other social position proxies, such as education level, Africans (especially women) have been historically compromised in terms of education and earning capacity under the South African apartheid laws [34,35,36,57,58]. What we know is that in South Africa, the majority are regarded to have no income. In fact, access to education in the country is also difficult. Hence, the large majority of South Africans struggle to sustain decent employment opportunities [57]. In some segments of the population in South Africa, fewer individuals achieve their senior certificates. For those who achieve it, few have access to tertiary institutions to pursue higher education [58]. Moreover, among those who manage to achieve higher education, many still do not have access to decent employment [57]. They therefore become confined/restricted to low-level or non-specialized low paying employment positions, which sometimes constrains their social position [34]. This is still currently reflected in the latest Quarterly Labor Force Survey (QLFS) [57], where it has been shown that more than half (or 51%) of African youth aged 18–24 have less access to higher education institutions. When probed, they have highlighted financial constraints as the barrier to access higher education [59]. These young South Africans end up in non-permanent or part time job positions that are offered in construction, mining and agricultural industries [57,58]. While there has been a steady loss of jobs in all employment professions in South Africa since 2017, the net job loss has mainly been driven by job loss in construction (142,000), mining (20,000) and agriculture (12,000) [57]. Moreover, according to the South African Living Conditions Survey (LCS) [34], 84.6%, 20.9%, 46,9% of White, Colored, and Indian/Asian South African households, respectively, fall within the highest social position quintile compared to 11.1% of African households. According to the similar survey, LCS published in 2015 [34], it appears as though non-Africans commanded high average incomes (444,446 ZAR [31,329 USD] for White South Africans and 271,621 ZAR [19,146 USD] for Indian/Asian South Africans per year). These are three and five times greater than the average incomes for Africans (estimated at about 92,893 ZAR [6548 USD] per year). Moreover, in the same survey, male-headed households were shown to secure an almost double (165,853 ZAR [11,691 USD]) average income compared to their female-headed household counterparts (98,911 ZAR [6972 USD]). These survey results clearly indicate that men, non-Africans, and those South Africans residing in male-headed households continue to be at an increased social position advantage compared to their female and African counterparts. As such, differences in obesity and disease prevalence between genders and ethnic groups in South Africa may be partly attributed to or mediated by these aforementioned social inequalities. 

Despite the strengths of the current research, there are limitations that need to be considered, amongst them being almost 50% of South Africans not correctly reporting their household income. Hence, we restricted our analyses of BMI versus household income to 1140 (50%) men and 2218 (53%) women. It is important to note that in South Africa, quantifying monthly income is difficult because the majority are unemployed. Some of the unemployed generate their income through non-formal or less formal businesses like street food vending or selling their produce from subsistence farming. For those who are employed, they are employed in the informal sector [57]. Nonetheless, the outcomes obtained from this study can be regarded as valid because in this analysis, similar social patterning outcomes were obtained across the BMI groups with or without data on household income. 

Another limitation is that a number of factors, including infectious diseases like HIV and genetics, could not be included in our study; these topics were beyond the scope of the current research. However, we acknowledge the influence of behavior (i.e., diet, physical activity, smoking and alcohol misuse) on underweight, overweight and obesity. It is increasingly being recognized that overfeeding and high maternal BMI during pregnancy can result in obesity in the offspring later in life [60]. Barrera et al. [61] showed that obesity actively promotes the increase of different chronic pathologies. In fact, Kruger et al. [62] also argued that the overconsumption of food high in simple sugars and saturated fats may cause glucose and lipid toxicity, respectively. All the aforementioned conditions are associated with insulin resistance. Insulin resistance fuels the pathogenesis of the co-morbid diseases of obesity, among them being type 2 diabetes and cardiovascular diseases. In terms of physical activity, fewer women in South Africa are active when compared to men [4]. In fact, only 42% of South African women have been found to be physically fit compared to almost two-thirds of men (62.4%) [4]. A small study on African women [16] showed that those women who are physically active tend to be lighter, have less overall body fat accumulation, have higher serum high density lipoprotein cholesterol (HDL-C) concentrations, and are more insulin sensitive compared to their less physically active counterparts. Furthermore, while the risk of high BMI is lower in those South Africans who smoke [6], cigarette smoking accounts for a large burden of preventable disease in South Africa, amongst them being the lung cancer, cardiovascular diseases and chronic obstructive pulmonary diseases [63]. Finally, there is a strong significant positive association between BMI and excessive alcohol consumption [64]. Hernandez-Rodas et al. [65] argued that dietary, nutritional interventions and lifestyle changes can contribute to prevention or mitigation to these health problems. Shifts are also needed to counter the marketing of calorie-dense and nutrient-poor products to the vulnerable populations of South Africa [66,67]. Healthy messaging, targeting the discretionary intake of salt and sugar and encouraging regular physical exercise, especially for girls and women in the country, is needed [62]. 

## 5. Conclusions

The current results suggest that the majority of South Africans fall on the overweight side of the BMI spectrum. African women are at a greater risk of obesity than non-African women and men in both ethnicities. Socioeconomic disadvantage increases the risks of underweight in African men. Being in any form of marriage/union in both genders increases the risk of overweight and obesity. While a higher income is associated with overweight and obesity in men, poor mental health is associated with underweight in this gender. These findings have public health implications, especially in the South African population that grapples with malnutrition (as indicated by the steady prevalence of underweight in African men and escalating prevalence of obesity in African women) and the rapidly growing NCD epidemic. Actions are therefore needed to prevent malnutrition among socioeconomic disadvantaged groups in the country, especially African men and women. A generally high prevalence of obesity among women and positive associations of education, employment and income with overweight and obesity call for investments into health literacy.

## Figures and Tables

**Table 1:** Body mass index of South African men (2254) and women (4170) from the SANHANES-1 study in 2012; by age.

Age Group (Years)	Underweight % (*n*)	Healthy BMI % (*n*)	Overweight % (*n*)	Obesity % (*n*)	Total *N*
15–24	9.9 (174)	61.1 (1069)	16.9 (295)	12.2 (213)	1 751
25–44	6.8 (141)	38.9 (806)	23.9 (494)	30.4 (629)	2 070
45–64	6.3 (119)	29.2 (549)	24.6 (462)	39.9 (751)	1 881
65+	6.8 (49)	33.4 (241)	25.5 (184)	34.4 (248)	722
Total	7.5 (483)	41.5 (2665)	22.3 (1435)	28.7 (1841)	6 424

**Table 2:** Age adjusted proportions of underweight, healthy weight, overweight and obesity among 2254 men from the SANHANES study in 2012; by indicators of socioeconomic position.

Socio-Demographic Factors	Men	Underweight Proportion (95% CI)	Healthy Weight Proportion (95% CI)	Overweight Proportion (95% CI)	Obese Proportion (95% CI)
	*N*				
Ethnicity:					
African	1109	11.9 (10.1, 13.9)	57.7 (54.9, 60.5)	18.7 (16.6, 21.1)	11.6 (9.8, 13.6)
Non-African	546	13.1 (10.4, 16.2)	50.9 (46.7, 55.0)	23.1 (19.8, 26.8)	12.9 (10.4, 15.9)
Education:					
No education	155	26.9 (18.4, 37.5)	38.1 (29.2, 48.0)	27.1 (20.6, 34.7)	7.8 (4.9, 12.2)
Grade 1–7	420	16.4 (13.0, 20.6)	57.9 (52.8, 62.9)	16.5 (13.5, 20.1)	9.1 (6.4, 12.7)
Grade 8–11	664	12.0 (9.7, 14.8)	57.0 (53.2, 60.8)	20.3 (17.2, 23.7)	10.6 (8.4, 13.4)
Grade 12	305	7.0 (4.0, 12.0)	52.5 (44.9, 60.0)	20.7 (15.1, 27.7)	19.7 (14.1, 26.9)
Higher	111	1.9 (0.5, 7.1)	39.4 (30.9, 48.5)	36.5 (28.2, 45.8)	22.2 (15.7, 30.5)
Employment:					
Not employed	1087	15.8 (13.7, 18.2)	56.5 (53.5, 59.5)	18.4 (16.2, 20.9)	9.3 (7.6, 11.2)
Employed	568	4.6 (3.3, 6.4)	58.4 (53.8, 62.9)	19.7 (16.3, 23.6)	17.3 (13.7, 21.5)
Marital status:					
Married/cohabiting	763	10.4 (6.7, 15.9)	49.5 (43.2, 55.8)	24.5 (20.1, 29.5)	15.6 (11.7, 20.6)
Never married	797	17.2 (13.7, 21.4)	60.7 (55.8, 65.4)	14.4 (11.1, 18.5)	7.6 (5.3, 10.9)
Divorced/separated/widowed	95	16.6 (8.0, 31.3)	56.7 (44.5, 68.1)	18.6 (10.9, 30.0)	8.1 (3.1, 19.3)
Poor mental health:					
No	1388	11.1 (9.6, 12.9)	56.1 (53.6, 58.7)	20.6 (18.6, 22.8)	12.1 (10.5, 13.9)
Yes	267	18.3 (13.9, 23.8)	55.0 (49.0, 60.8)	16.0 (12.4, 20.5)	10.6 (7.7, 14.5)
Household Income *:					
No income	363	14.7 (10.8, 20.0)	56.4 (49.8, 62.7)	14.0 (10.5, 18.5)	14.8 (10.2, 21.0)
Low income	589	13.2 (10.5, 16.4)	58.0 (53.8, 62.0)	19.3 (16.4, 22.6)	9.5 (7.3, 12.2)
Medium/high income	188	2.7 (1.1, 6.6)	46.1 (38.0, 54.3)	27.6 (20.7, 35.8)	23.6 (17.4, 31.1)

* Restricted to 1140 men and 2218 women with additional data on income available. Underweight, healthy BMI, overweight and obesity were defined as BMI levels of <5th percentile and <18.5 kg/m^2^, 5–84.9th percentile and 18.5–24.9 kg/m^2^, 85–94.9th percentile and 25–29.9 kg/m^2^, and ≥95th percentile and ≥30 kg/m^2^ for children and adults, respectively (CDC, 24/7) [45].

**Table 3:** Age adjusted proportions of underweight, healthy weight, overweight and obesity among 4170 women from the SANHANES study in 2012; by indicators of socioeconomic position.

Socio-Demographic Factors	Women	Underweight Proportion (95% CI)	Healthy Weight Proportion (95% CI)	Overweight Proportion (95% CI)	Obese Proportion (95% CI)
	*N*				
Ethnicity:					
African	2120	4.2 (3.5, 5.2)	31.3 (29.4, 33.2)	23.3 (21.5, 25.1)	41.1 (39.2, 43.2)
Non-African	984	4.8 (3.6, 6.3)	34.2 (31.4, 37.2)	26.6 (23.9, 29.5)	34.3 (31.4, 37.2)
Education:					
No education	350	8.3 (4.6, 14.6)	36.7 (29.3, 44.9)	21.4 (16.4, 27.4)	33.5 (26.6, 41.2)
Grade 1–7	760	6.7 (4.8, 9.2)	33.3 (29.5, 37.3)	23.7 (20.5, 27.3)	36.2 (32.6, 40.0)
Grade 8–11	1185	2.8 (2.0, 3.9)	27.4 (25.0, 30.0)	25.9 (23.3, 28.7)	43.9 (41.0, 46.8)
Grade 12	631	3.2 (2.1, 4.8)	36.1 (31.3, 41.2)	22.8 (19.0, 27.1)	37.9 (32.9, 43.1)
Higher	178	2.3 (0.8, 6.2)	24.2 (18.5, 30.9)	22.7 (17.0, 29.6)	50.9 (43.8, 57.8)
Employment:					
Not employed	2406	4.6 (3.8, 5.5)	33.6 (31.8, 35.5)	23.4 (21.7, 25.2)	38.3 (36.4, 40.2)
Employed	698	4.4 (2.0, 9.3)	22.1 (18.2, 26.5)	29.2 (23.5, 35.8)	44.2 (39.3, 49.2)
Marital status:					
Married/cohabiting	1244	3.8 (2.6, 5.5)	32.6 (30.0, 35.7)	24.4 (21.9, 27.2)	39.1 (36.4, 41.9)
Never married	1407	5.1 (3.9, 6.6)	35.5 (32.7, 38.4)	22.5 (20.0, 25.1)	36.9 (34.0, 40.0)
Divorced/separated/widowed	453	2.3 (0.9, 5.6)	39.0 (28.6, 50.5)	22.3 (13.5, 34.6)	36.4 (31.3, 41.7)
Poor mental health:					
No	2425	4.0 (3.3, 4.8)	32.9 (31.1, 34.7)	24.3 (22.7, 26.1)	38.7 (36.9, 40.6)
Yes	679	6.1 (4.4, 8.3)	30.0 (26.6, 33.6)	24.8 (21.6, 28.4)	39.1 (35.6, 42.6)
Household Income *:					
No income	817	4.3 (3.1, 5.8)	30.3 (26.5, 34.3)	21.9 (18.8, 25.4)	43.5 (39.3, 47.8)
Low income	1235	4.4 (3.4, 5.9)	29.0 (26.4, 31.7)	23.9 (21.5, 26.5)	42.6 (39.9, 45.4)
Medium/high income	166	2.6 (0.8, 7.9)	23.2 (15.4, 33.5)	28.9 (20.6, 38.9)	45.2 (38.4, 52.2)

* Restricted to 1140 men and 2218 women with additional data on income available. Underweight, healthy BMI, overweight and obesity were defined as BMI levels of <5th percentile and < 18.5 kg/m^2^, 5–84.9th percentile and 18.5–24.9 kg/m^2^, 85–94.9th percentile and 25–29.9 kg/m^2^, and ≥95th percentile and ≥ 30 kg/m^2^ for children and adults, respectively (CDC, 24/7) [45].

**Table 4:** Relative risk of underweight, overweight and obesity among 1655 men from the SANHANES study in 2012; by indicators of socioeconomic position (minimally and fully adjusted odds ratios from multinomial regression).

Socio-Demographic Factors	Men							
	Model 1:				Model 2:			
	Underweight	Healthy Weight	Overweight	Obesity	Underweight	Healthy Weight	Overweight	Obesity
	Age-adjusted OR (95% CI)	Reference (*p*-value)	Age-adjusted OR (95% CI)	Age-adjusted OR (95% CI)	Fully Adjusted OR (95% CI)	Reference (*p*-value)	Fully Adjusted OR (95% CI)	Fully Adjusted OR (95% CI)
Ethnicity:								
African	1	1 (0.066)	1	1	1	1 (0.027)	1	1
Non-African	1.25 (0.90, 1.73)		1.40 (1.07, 1.84)	1.28 (0.92, 1.77)	1.66 (1.17, 2.34)		1.26 (0.95, 1.68)	1.11 (0.78, 1.57)
Education:								
No-education	1	1 (< 0.001)	1	1	1	1 (< 0.001)	1	1
Grade 1–7	0.73 (0.43, 1.23)		1.08 (0.66, 1.77)	0.75 (0.41, 1.39)	0.74 (0.43, 1.26)		1.07 (0.65, 1.77)	0.77 (0.41, 1.43)
Grade 8–11	0.47 (0.27, 0.81)		1.32 (0.80, 2.16)	1.06 (0.58, 1.92)	0.45 (0.26, 0.78)		1.20 (0.72, 1.99)	0.98 (0.53, 1.81)
Grade 12	0.24 (0.12, 0.48)		1.57 (0.89, 2.76)	2.13 (1.11, 4.09)	0.24 (0.12, 0.49)		1.44 (0.81, 2.58)	1.92 (0.98, 3.76)
Higher	0.12 (0.03, 0.53)		3.54 (1.88, 6.68)	3.35 (1.61, 6.96)	0.14 (0.03, 0.62)		3.13 (1.64, 5.98)	2.73 (1.29, 5.79)
Employment:								
Not employed	1	1 (< 0.001)	1	1	1	1 (< 0.001)	1	1
Employed	0.39 (0.25, 0.59)		1.35 (1.00, 1.81)	2.02 (1.43, 2.84)	0.44 (0.29, 0.68)		1.10 (0.81, 1.50)	1.63 (1.14, 2.35)
Marital status:								
Married/cohabiting	1	1 (< 0.001)	1	1	1	1 (< 0.001)	1	1
Never married	1.49 (0.98, 2.24)		0.46 (0.32, 0.65)	0.37 (0.24, 0.57)	1.27 (0.83, 1.95)		0.49 (0.34, 0.70)	0.42 (0.27, 0.66)
Divorce/separated/widowed	1.40 (0.72, 2.72)		0.66 (0.38, 1.13)	0.46 (0.22, 0.98)	1.34 (0.68, 2.64)		0.67 (0.39, 1.16)	0.52 (0.24, 1.12)
Poor mental health:								
No	1	1 (0.014)	1	1	1	1 (0.089)	1	1
Yes	1.74 (1.19, 2.55)		0.85 (0.59, 1.21)	0.98 (0.64, 1.50)	1.63 (1.09, 2.42)		0.96 (0.66, 1.40)	1.21 (0.78, 1.90)

Model 1: Minimally adjusted. Adjusted for age in seven categories (15–18, 19–24, 25–34, 35–44, 45–54, 55–64, and 65+ years). Model 2: Fully adjusted. Adjusted for age and all other social and health variables included in the table. *p*-value from test for heterogeneity: *p*-value for interaction test between gender and ethnicity *p* = 0.039, gender and education *p* < 0.001, gender and employment *p* = 0.012, and gender and marital status *p* = 0.001. Underweight, healthy BMI, overweight and obesity were defined as BMI levels of <5th percentile and <18.5 kg/m^2^, 5–84.9th percentile and 18.5–24.9 kg/m^2^, 85–94.9th percentile and 25–29.9 kg/m^2^, and ≥95th percentile and ≥30 kg/m^2^ for children and adults, respectively (CDC, 24/7) [45].

**Table 5:** Relative risk of underweight, overweight and obesity among 3104 women from the SANHANES study in 2012; by indicators of socioeconomic position (minimally and fully adjusted odds ratios from multinomial regression).

Socio-Demographic Factors	Women							
	Model 1:				Model 2:			
	Underweight	Healthy Weight	Overweight	Obesity	Underweight	Healthy Weight	Overweight	Obesity
	Age-adjusted OR (95% CI)	Reference (*p*-value)	Age-adjusted OR (95% CI)	Age-adjusted OR (95% CI)	Fully Adjusted OR (95% CI)	Reference (*p*-value)	Fully Adjusted OR (95% CI)	Fully Adjusted OR (95% CI)
Ethnicity:								
African	1	1 (0.002)	1	1	1	1 (< 0.001)	1	1
Non-African	1.02 (0.70, 1.50)		1.03 (0.84, 1.26)	0.74 (0.61, 0.89)	1.26 (0.85, 1.87)		0.93 (0.76, 1.15)	0.64 (0.52, 0.78)
Education:								
No-education	1	1 (< 0.001)	1	1	1	1 (< 0.001)	1	1
Grade 1–7	0.73 (0.41, 1.32)		1.28 (0.88, 1.86)	1.26 (0.91, 1.74)	0.69 (0.38, 1.27)		1.28 (0.88, 1.87)	1.35 (0.97, 1.88)
Grade 8–11	0.34 (0.18, 0.66)		1.82 (1.24, 2.67)	2.15 (1.54, 3.00)	0.32 (0.16, 0.62)		1.82 (1.24, 2.69)	2.43 (1.72, 3.42)
Grade 12	0.36 (0.18, 0.72)		1.27 (0.84, 1.91)	1.36 (0.95, 1.96)	0.33 (0.16, 0.68)		1.24 (0.82, 1.88)	1.51 (1.04, 2.19)
Higher	0.34 (0.11, 1.08)		1.55 (0.90, 2.67)	2.31 (1.44, 3.71)	0.33 (0.10, 1.05)		1.42 (0.82, 2.48)	2.45 (1.51, 3.97)
Employment:								
Not employed	1	1 (0.005)	1	1	1	1 (0.005)	1	1
Employed	1.02 (0.61, 1.70)		1.50 (1.17, 1.92)	1.38 (1.10, 1.73)	1.12 (0.66, 1.88)		1.54 (1.20, 1.98)	1.38 (1.10, 1.75)
Marital status:								
Married/cohabiting	1	1 (0.083)	1	1	1	1 (0.068)	1	1
Never married	1.17 (0.74, 1.85)		0.75 (0.59, 0.95)	0.78 (0.63, 0.97)	1.18 (0.75, 1.85)		0.77 (0.60, 0.97)	0.76 (0.61, 0.95)
Divorce/separated/widowed	0.68 (0.34, 1.38)		0.86 (0.62, 1.20)	0.93 (0.70, 1.24)	0.64 (0.31, 1.30)		0.84 (0.60, 1.17)	0.89 (0.67, 1.20)
Poor mental health:								
No	1	1 (0.091)	1	1	1	1 (0.079)	1	1
Yes	1.71 (1.13, 2.58)		1.12 (0.88, 1.42)	1.14 (0.91, 1.41)	1.74 (1.14, 2.64)		1.16 (0.91, 1.48)	1.13 (0.91, 1.42)

Model 1: Minimally adjusted. Adjusted for age in seven categories (15–18, 19–24, 25–34, 35–44, 45–54, 55–64, and 65+ years). Model 2: Fully adjusted. Adjusted for age and all other social and health variables included in the table. *p*-value from test for heterogeneity: *p*-value for interaction test between gender and ethnicity *p* = 0.039, gender and education *p* < 0.001, gender and employment *p* = 0.012, and gender and marital status *p* = 0.001. Underweight, healthy BMI, overweight and obesity were defined as BMI levels of <5th percentile and <18.5 kg/m^2^, 5–84.9th percentile and 18.5–24.9 kg/m^2^, 85–94.9th percentile and 25–29.9 kg/m^2^, and ≥95th percentile and ≥30 kg/m^2^ for children and adults, respectively (CDC, 24/7) [45].

**Table 6:** Relative risk of underweight, overweight and obesity among 1140 men and 2218 women from the SANHANES study in 2012; by income (minimally and fully adjusted odds ratios from multinomial regression).

**Socio-Economic Factors**	**Men**							
	**Model 1:**				**Model 2:**			
	**Underweight**	**Healthy Weight**	**Overweight**	**Obesity**	**Underweight**	**Healthy Weight**	**Overweight**	**Obesity**
	**Age-adjusted OR (95% CI)**	**Reference** **(*p*-value)**	**Age-adjusted OR (95% CI)**	**Age-adjusted OR (95% CI)**	**Fully Adjusted OR (95% CI)**	**Reference** **(*p*-value)**	**Fully Adjusted OR (95% CI)**	**Fully Adjusted OR (95% CI)**
Household Income:								
No income	1.18 (0.77, 1.81)	1 (< 0.001)	0.85 (0.56, 1.29)	1.50 (0.92, 2.44)	0.99 (0.59, 1.67)	1 (0.004)	0.76 (0.47, 1.24)	1.77 (1.00, 3.13)
Low income	1		1	1	1		1	1
Medium/high income	0.25 (0.10, 0.65)		2.04 (1.33, 3.13)	3.71 (2.28, 6.02)	0.43 (0.16, 1.16)		1.48 (0.90, 2.42)	2.14 (1.22, 3.75)
**Socio-Economic Factors**	**Women**							
	**Model 1:**				**Model 2:**			
	**Underweight**	**Healthy Weight**	**Overweight**	**Obesity**	**Underweight**	**Healthy Weight**	**Overweight**	**Obesity**
	**Age-adjusted OR (95% CI)**	**Reference** **(*p*-value)**	**Age-adjusted OR (95% CI)**	**Age-adjusted OR (95% CI)**	**Fully Adjusted OR (95% CI)**	**Reference** **(*p*-value)**	**Fully Adjusted OR (95% CI)**	**Fully Adjusted OR (95% CI)**
Household Income:								
No income	1.04 (0.65, 1.68)	1 (0.136)	0.91 (0.70, 1.18)	0.95 (0.75, 1.20)	1.18 (0.69, 2.02)	1 (0.649)	1.08 (0.80, 1.45)	1.07 (0.82, 1.39)
Low income	1		1	1	1		1	1
Medium/high income	0.87 (0.29, 2.62)		1.56 (0.94, 2.59)	1.78 (1.13, 2.80)	1.10 (0.32, 3.71)		1.51 (0.85, 2.68)	1.63 (0.97, 2.74)

Model 1: Minimally adjusted. Adjusted for age in seven categories (15–18, 19–24, 25–34, 35–44, 45–54, 55–64, and 65+ years). Model 2: Fully adjusted. Adjusted for age, ethnicity, education, marital status, employment and mental health status. *p*-value from test for heterogeneity: *p*-value for interaction test between gender and income *p* = 0.042. Underweight, healthy BMI, overweight and obesity were defined as BMI levels of <5th percentile and <18.5 kg/m^2^, 5–84.9th percentile and 18.5–24.9 kg/m^2^, 85–94.9th percentile and 25–29.9 kg/m^2^, and ≥95th percentile and ≥30 kg/m^2^ for children and adults, respectively (CDC, 24/7) [45].

**Table 7:** Relative risks of overweight or obesity among 1655 men and 3104 women from the SANHANES study in 2012; by indicators of socioeconomic position (results from logistic regression).

Socio-Demographic Characteristics	Men		Women	
	*N* = 1655		*N* = 3104	
	Age-adjusted OR (95% CI)	Fully Adjusted OR (95% CI)	Age-adjusted OR (95% CI)	Fully Adjusted OR (95% CI)
Ethnicity:				
African	1	1	1	1
Non-African	1.30 (1.04, 1.62)	1.11 (0.87, 1.41)	0.85 (0.72, 1.00)	0.73 (0.62, 0.87)
*p*-value	0.024	0.402	0.053	< 0.001
Education:				
No education	1	1	1	1
Grade 1–7	1.03 (0.69, 1.55)	1.03 (0.68, 1.56)	1.33 (1.00, 1.77)	1.40 (1.05, 1.86)
Grade 8–11	1.44 (0.96, 2.16)	1.31 (0.86, 1.99)	2.32 (1.73, 3.11)	2.50 (1.85, 3.38)
Grade 12	2.32 (1.46, 3.68)	2.07 (1.28, 3.33)	1.53 (1.11, 2.10)	1.61 (1.17, 2.23)
Higher	4.70 (2.73, 8.08)	3.88 (2.22, 6.77)	2.29 (1.50, 3.50)	2.28 (1.48, 3.51)
*p*-value heterogeneity	< 0.001	< 0.001	< 0.001	< 0.001
Employment:				
Not employed	1	1	1	1
Employed	1.85 (1.45, 2.35)	1.44 (1.12, 1.87)	1.43 (1.17, 1.74)	1.43 (1.17, 1.76)
*p*-value	< 0.001	0.005	< 0.001	< 0.001
Marital status:				
Married/cohabiting	1	1	1	1
Never married	0.39 (0.30, 0.53)	0.44 (0.33, 0.60)	0.76 (0.63, 0.91)	0.75 (0.62, 0.91)
Divorced/separated/widowed	0.55 (0.35, 0.87)	0.58 (0.36, 0.93)	0.95 (0.73, 1.23)	0.92 (0.71, 1.20)
*p*-value heterogeneity	< 0.001	< 0.001	0.013	0.013
Poor mental health:				
No	1	1	1	1
Yes	0.79 (0.59, 1.06)	0.95 (0.69, 1.29)	1.05 (0.87, 1.27)	1.06 (0.88, 1.29)
*p*-value	0.121	0.729	0.618	0.536

Minimally adjusted: Adjusted for age in seven categories (15–18, 19–24, 25–34, 35–44, 45–54, 55–64, and 65+ years). Fully adjusted: Adjusted for age and all other social and health variables included in the table. Examination of heterogeneity, as indicated by an interaction between: i) gender and ethnicity (*p* = 0.004), ii) gender and education (*p* < 0.001), and iii) gender and marital status (*p* = 0.003).
